# A case report of an asymptomatic late term abdominal pregnancy with a live birth at 41 weeks of gestation

**DOI:** 10.1186/s13104-016-1844-6

**Published:** 2016-01-19

**Authors:** Mercy Nkuba Nassali, Tadele Melese Benti, Moreri Bandani-Ntsabele, Elly Musinguzi

**Affiliations:** Department of Obstetrics and Gynaecology, Faculty of Medicine, University of Botswana, Private Bag 00713, Gaborone, Botswana; Department of Obstetrics and Gynaecology, Princess Marina Hospital, P.O. Box 258, Gaborone, Botswana

**Keywords:** Asymptomatic, Advanced, Abdominal pregnancy, Live birth

## Abstract

**Background:**

Despite advances in diagnostic imaging and focused antenatal care, cases of undiagnosed abdominal pregnancies at term are still reported in obstetric practice. It is atypical and very rare for a patient to be asymptomatic late in pregnancy and for the pregnancy to result in a live birth with no evidence of intrauterine growth restriction despite the unfavourable implantation site. This late term asymptomatic presentation despite routine antenatal care demonstrates a diagnostic challenge.

**Case presentation:**

We report a case of a 26 year old Primigravida with an asymptomatic and undiagnosed abdominal pregnancy carried beyond 41 weeks of gestation espite routine antenatal care and serial ultrasound reports. She presented for a routine antenatal care visit at 41 weeks of gestation. Induction of labour was initiated due to the late term gestation but was unsuccessful. At this point the fetus developed severe tachycardia and CTG confirmed persistent non-reassuring foetal heart rate patterns. The mother was then prepared for an emergency caesarean delivery. Abdominal pregnancy was only diagnosed at laparotomy where a term male baby weighing 3108 g was delivered with an Apgar Score of 7 and 8 at 1 and 5 min respectively. The placenta which was implanted into the omentum, ileal mesentery and extending to the pouch of Douglas was removed following active bleeding from its detached margins. She was transfused with two units of blood and four units of fresh frozen plasma. Postoperative morbidity was minimal with transient paralytic ileus on the second post-operative day. Her recovery was otherwise uneventful and she was discharged on the seventh post-operative day in good condition. The neonate developed meconium aspiration syndrome and passed away on the 2nd day of life despite having undergone standard care. A post-mortem examination was not performed because the family did not consent to the procedure. Follow up of the mother at 2, 6 weeks and 6 months postpartum was uneventful.

**Conclusions:**

This atypical presentation of an asymptomatic abdominal pregnancy carried tolate term and only diagnosed at laparotomy despite routine antenatal care demonstrates a significant lapse in diagnosis. Clinicians and radiologists must always bear this possibility in mind during routine client evaluation. Skills training in Obstetric ultrasound and in the clinical assessment of obstetric patients should emphasize features suggestive of abdominal pregnancy. This will improve diagnosis, ensure appropriate management and minimise complications. Immediate termination of pregnancy can be offered if the diagnosis is made before 20 weeks of gestation. Patients diagnosed with advanced abdominal pregnancies and are stable can be monitored under close surveillance and delivered at 34 weeks of gestation after lung maturity is achieved. Although removal of the placenta carries a higher risk of haemorrhage, a partially detached placenta can be delivered with minimal morbidity and a good maternal outcome. Given the documented low survival rates of neonates in such cases, neonatal units must be adequately equipped and staffed to support them. Post-mortem examination is important to confirm cause of death and exclude other complications and congenital anomalies. Communities need to be educated about the importance of this procedure.

## Background

Abdominal pregnancy is a rare condition resulting from implantation of the embryo in the peritoneal cavity external to the uterine cavity, fallopian tubes and broad ligament. It is referred to as an advanced abdominal pregnancy when it has gone beyond 20 weeks of gestation with signs of a foetus living or having lived and developed in the mother’s peritoneal cavity. Although primary peritoneal implantation remains a rare possibility, this occurrence is more often a result of either a tubal abortion or a self-limited tubal rupture with secondary intra-peritoneal Implantation. In the USA, the incidence of abdominal pregnancy is 1 per 10,000 live births [1]. Abdominal pregnancy accounts for approximately 1 % of ectopic pregnancies and ectopic pregnancies account for 1–2 % of all pregnancies [2]. A late term abdominal pregnancy with a viable foetus is an even rarer phenomenon with few reported cases in the literature [3].

Though rare, the challenge it poses to obstetric practice due to the associated high maternal and perinatal morbidity and mortality makes it worth noting. Despite advances in diagnostic imaging, preoperative diagnosis remains a challenge with only 20–40 % of cases diagnosed pre-operatively [4]. Even in developed health systems, diagnosis is often made intra-operatively. A review of 5221 cases of abdominal pregnancy in the USA found that only 11 % were diagnosed pre-operatively [1].

First trimester ultrasound scan is important because the likelihood of missing the diagnosis is greater in the second and third trimesters. Ultrasound diagnostic error is estimated at 50–90 % in several case series [5]. A classic feature of absent myometrium between the maternal bladder and the foetus guides ultrasound diagnosis of abdominal pregnancy [6]. Other diagnostic features include an empty uterus, an ill-defined placenta, oligohydramnios and an abnormal foetal lie [7]. Better prospects are reported with magnetic resonance imaging (MRI) [8]. Elevated levels of α fetoprotein levels may also guide diagnosis [9].

Clinical features of abdominal pregnancy range from an asymptomatic presentation to symptoms such as generalised abdominal pain, foetal malpresentation, unexplained foetal demise, acute abdomen and failed induction of labour. Correct diagnosis therefore calls for a high index of suspicion [10].

Traditionally, the practise has been to leave the placenta in situ to undergo spontaneous resorption. Methotrexate therapy may be used to enhance the process. Leaving the placenta in situ minimises the risk of haemorrhage but puts the patient at risk for necrosis, pelvic abscess and wound dehiscence. Several case reports have described removal of the placenta with relatively good outcomes [2]. This decision should however be individualised depending on the colonisation site [11].

Common foetal complications include malformations, intrauterine growth restriction and foetal demise [12]. Overall the chance of having a live birth in a case of abdominal pregnancy is estimated at 10–20 % [5]. Congenital anomalies occur in up to 40 % of cases with about half of these surviving through the 1st week of life [2, 13]. Several reports have cited high perinatal mortality rates ranging from 80 to 95 % [5, 14]. This case report describes diagnostic challenges and the implications of poor clinical skills in obstetric care.

## Case presentation

We present a 26 year African female, Primigravida with an undiagnosed asymptomatic abdominal pregnancy at 41 1/7 weeks of gestation. She registered for antenatal care at 14 weeks of gestation at a Primary Health Care clinic where she had attended 12 visits. She was then referred to the national referral hospital for further management because she was beyond her due date for delivery. The pregnancy was uneventful except for the mild to moderate anaemia which was managed with oral Iron therapy. The haemoglobin level was 8.7 g/dl at 14 weeks, 9.0 g/dl at 28 weeks and 10.3 g/dl at 36 weeks and 9.9 g/dl at 41 weeks respectively. She was also diagnosed to be human immune deficiency virus (HIV) positive at her first antenatal care (ANC) visit and highly active antiretroviral therapy (HAART) was initiated. The blood group was A Rhesus positive and VDRL screening for syphilis was non-reactive. The previous medical, surgical, gynaecological and social history was unremarkable.

Although the first ultrasound scan was requested at 24 weeks at the primary health care clinic, this was only accessed at 36 weeks of gestation at the national referral hospital. The ultrasound report revealed a singleton intrauterine foetus, in cephalic presentation, at 34 weeks of gestation and with an estimated foetal weight of 2376 g. The placenta was reported to be fundal posterior and the cervix was closed. On admission, the patient appeared well with no reported pallor and her vital signs were normal. The abdomen was symmetrically distended with a fundal height of 37 cm. There were no areas of tenderness and foetal parts were not easily palpable. The foetal presentation was cephalic, the lie was longitudinal and the descent of the presenting part was 5/5. The foetal heart rate was 142 bpm and regular. Pelvic examination revealed a posterior, firm, uneffaced and closed cervix. The station of the presenting part was at −3 and the pelvis was deemed adequate. A second ultrasound on admission by a different sonographer similarly reported a single, live, intrauterine foetus, at 38 weeks gestation, in cephalic presentation, with adequate volume of liquor and an estimated foetal weight of 3271 g. The placenta was posterior and the cervical os was closed. Cervical ripening was initiated with oral Misoprostol 25 μg orally 2 hourly. Assessment after the sixth dose revealed no improvement in the Bishop Score. The foetus developed tachycardia and CTG confirmed a persistent baseline tachycardia of 180 bpm with decreased beat to beat variability. This pattern did not improve despite the instituted resuscitation measures; oxygen therapy at 8 l/min, a left lateral maternal tilt and a litre of Normal Saline infusion. Maternal vital signs remained stable and the patient reported no alteration in foetal movements, no vaginal bleeding and no abnormal vaginal discharge. She did however report mild abdominal discomfort. There were no palpable uterine contractions and no localised tenderness was elicited. A decision to perform an emergency caesarean delivery was then made due to the non-reassuring foetal heart rate pattern. Under spinal anaesthesia and following standard aseptic technique, the abdomen was opened through Pfannenstiel incision.

### Intraoperative findings

An intact gestational sac was found in the peritoneal cavity with grade 3 meconium stained liquor. There was no haemoperitoneum. A male baby was delivered with an Apgar Score of 7 and 8 at 1 and 5 minutes respectively, weighing 3108 g. No gross congenital abnormality was reported. The baby aspirated meconium stained liquor but responded to suction followed by bag and mask resuscitation.^1^ He was then admitted to the neonatal unit for on-going care. The uterus was slightly enlarged at about 12 week size. The fallopian tubes and ovaries were normal. The placenta had implanted into the omentum, ileal mesentery and extended to the pouch of Douglas. A 400 g placenta was delivered following partial detachment that resulted in active bleeding from the margins. Haemostasis was eventually achieved; the estimated blood loss was 2000 ml and the patient became hypotensive intra-operatively with blood pressure levels dropping as low as 80/40 mmHg. She was successfully resuscitated with crystalloids, two units of packed red blood cells and 4 units of fresh frozen plasma.

### Post-operative follow up

The patient recovered well postoperatively and was ambulant by day 2. The post transfusion haemoglobin level was 8.9 g/dl. She developed a transient paralytic ileus on the second postoperative day which resolved with conservative management. She was discharged on day 7 in a satisfactory condition. Follow up at 2, 6 weeks and 6 months postpartum was unremarkable.

### Infant follow up

The baby seemed to stabilise initially but on the 2nd day of life his condition deteriorated with progressively worsening severe respiratory distress. This did not respond to management in the neonatal unit and the baby subsequently died. The cause of death was presumed to be meconium aspiration pneumonia. A post mortem examination was not performed because the family did not give consent.

## Discussion

The case above evaded antepartum diagnosis as described in several reports where only one in ten abdominal pregnancies is diagnosed preoperatively [1]. Eleje et al. similarly reports a post-dated asymptomatic abdominal pregnancy scheduled for elective caesarean section for placenta praevia only to be diagnosed at laparotomy [3]. This patient’s diagnosis was only made intra-operatively despite the early ANC booking, the 12 antenatal visits, two ultrasound scans by independent sonographers at 36 and 41 weeks of gestation and evaluation by the obstetric team before and during induction of labour. This scenario points to a failure in clinical skills training and/or lack of on-going professional development. Although Allibone et al. describe guidelines for improved Diagnosis [7], ultrasound diagnosis error in a series of case reports has been found to range from 50 to 90 % [5]. Inadequate skills training in obstetric ultrasound can result in failure to detect such asymptomatic cases. The major setback is that clinicians do not bear in mind the possibility of this rare condition. One case report demonstrates visualisation of an intrauterine balloon of a Foley’s catheter on ultrasound as an additional aid to improving diagnosis [15]. A focused early first trimester ultra sound and magnetic resonance imaging improve diagnosis. MRI is also used to assess the degree of placental adherence and determine preoperatively whether the placenta can be safely delivered or should be left in situ [8]. These technologies are however not readily available in resource limited settings. The lack of good obstetric clinical skills similarly led to failure in diagnosis. Focused clinical evaluation of every client (symptomatic or asymptomatic) with the likelihood of abdominal pregnancy in mind will improve diagnosis. Clinical features suggestive of abdominal pregnancy include; easily palpable foetal parts, tenderness, foetal malpresentation and a small for date pregnancy due to growth restriction or reduced liquor volume. Good clinical acumen, continuous professional development on such rare cases coupled with a high index of suspicion should improve diagnosis more so in resource limited settings. Failed induction is a recognised feature of abdominal pregnancy and should be a warning sign to re-evaluate a patient [10]. In this case, the pregnancy was carried beyond 41 weeks gestation due to failure in diagnosis. Consensus in obstetric practise is to terminate the pregnancy if diagnosed prior to 20 weeks. Conservative management can be considered if abdominal pregnancy is diagnosed after 20 weeks of gestation and delivery scheduled at 34 weeks of gestation [11]. This option is only feasible where a mother is well informed of the risks and benefits, is stable and is under close surveillance [14]. She must be hospitalised with 24 h access to anaesthesia, surgery and blood products. Growth restriction or congenital anomalies must be excluded and the placental implantation should not extend to the upper abdomen [5]. The new-born developed the meconium aspiration syndrome and died after 2 days. Overall perinatal mortality in abdominal pregnancy is up to 70–95 % [9, 14]. Although meconium aspiration syndrome can be life threatening, failure to support the new-born may point to limitations in the ability of the local neonatal units in handling the critically ill new-borns. Better staffing, vigilance and improved resources for neonatal units in the local setting will help save more neonates. A post mortem examination to confirm the cause of death and exclude other complications or congenital anomalies was not possible because the family did not consent to the procedure. There is need for community education about the importance of post mortem. Even when diagnosis and interventions are timely, abdominal pregnancy still carries a high risk for maternal morbidity and mortality with the most common complication being haemorrhage. The patient lost approximately 2 litres of blood and this necessitated transfusion. Removal of the placenta though associated with higher blood loss, may have better maternal outcomes in well selected cases [16].

Alternative interventions include methotrexate therapy and uterine artery embolization. Arterial embolization performed pre-operatively minimises blood loss [17]. Placental vascular embolization facilitates resorption of a placenta that is left in situ [18]. The procedure however, predisposes the mother to ileus, sepsis and intestinal perforation. The decision whether to remove the placenta or leave it in situ should therefore be considered on a case by case basis following careful assessment of the implantation site.

## Conclusions

An asymptomatic abdominal pregnancy carried to late term that is only identified at laparotomy despite routine antenatal and intrapartum care, demonstrates a significant lapse in diagnosis. Clinicians and radiologists must always consider the likelihood of this condition during routine patient evaluation. Skills training in Obstetric ultrasound and in clinical assessment of obstetric patients should emphasize features suggestive of abdominal pregnancy to arrive at an appropriate diagnosis. This in turn will ensure judicious management of such cases and minimise complications. If a diagnosis of abdominal pregnancy is made before 20 weeks of gestation, the patient can be offered termination of pregnancy. Advanced abdominal pregnancy can be managed expectantly under very close surveillance and delivery scheduled at 34 weeks of gestation following induction of lung maturity with Betamethasone. Removal of the placenta in well selected cases may have better maternal outcomes. Neonatal units should be well equipped and facilitated to support such critically ill new-borns. Post-mortem is necessary to confirm cause of death and exclude other co-morbidities or congenital anomalies. Communities should be educated about the importance of this procedure.

## Consent

Written informed consent was obtained from the patient for the publication of this case report.

## Abbreviations

ANC: antenatal care
CTG: cardiotocograph
HAAR: highly active antiretroviral therapy
Hb: haemoglobin
HIV: human immune deficiency virus
IV: intravenous
MAS: meconium aspiration syndrome
MRI: magnetic resonance imaging
USA: United States of America
USS: ultrasound

## Footnotes

1 Although research reports had shown no benefit of suction among non-vigorous babies born through meconium stained liquor, hospital practise and guidelines at time of this delivery (1 February 2014) had not been updated. The 2015 updated guidelines by the International Liaison Committee on Resuscitation (ILCOR) do not recommended the practise.
